# Towards inclusive learning environments in post-graduate medical education: stakeholder-driven strategies in Dutch GP-specialty training

**DOI:** 10.1186/s12909-024-05521-z

**Published:** 2024-05-17

**Authors:** N.M. van Moppes, M. Nasori, J. Bont, J.M. van Es, M.R.M. Visser, M.E.T.C. van den Muijsenbergh

**Affiliations:** 1 Amsterdam UMC location University of Amsterdam, Department of General Practice and Public Health Research Institute, Meibergdreef 9, Amsterdam, 1105 AZ The Netherlands; 2 Department of General Practice, Radboud University Medical Center, Nijmegen, The Netherlands; 3 Pharos, centre of expertise on health disparities, Utrecht, The Netherlands

**Keywords:** Inclusive Medical Education, Stakeholder-driven Strategies, GP-specialty Training

## Abstract

**Background:**

A recent study found that ethnic minority General Practice (GP)-trainees receive more negative assessments than their majority peers. Previous qualitative research suggested that learning climate-related factors play a pivotal role in unequal opportunities for trainees in post-graduate medical settings, indicating that insufficient inclusivity had put minority students at risk of failure and dropout.

**Study objectives:**

We aimed to develop broadly supported strategies for an inclusive learning climate in Dutch GP-specialty training.

**Methods:**

We employed Participatory Action Research (PAR)-methods, incorporating Participatory Learning and Action (PLA)-techniques to ensure equal voices for all stakeholders in shaping Diversity, Equity, and Inclusion (DEI)-strategies for GP-specialty training. Our approach engaged stakeholders within two pilot GP-specialty training institutes across diverse roles, including management, support staff, in-faculty teachers, in-clinic supervisors, and trainees, representing ethnic minorities and the majority population. Purposeful convenience sampling formed stakeholder- and co-reader groups in two Dutch GP-specialty training institutes. Stakeholder discussion sessions were based on experiences and literature, including two relevant frameworks, and explored perspectives on the dynamics of potential ethnic minority trainees’ disadvantages and opportunities for inclusive strategies. A co-reader group commented on discussion outcomes. Consequently, a management group prioritized suggested strategies based on expected feasibility and compatibility.

**Results:**

Input from twelve stakeholder group sessions and thirteen co-readers led to implementation guidance for seven inclusive learning environment strategies, of which the management group prioritized three:

• Provide DEI-relevant training programs to all GP-specialty training stakeholders;

• Appoint DEI ambassadors in all layers of GP-specialty training;

• Give a significant voice to minority GP-trainees in their education.

**Conclusion:**

The study’s participatory approach engaged representatives of all GP-specialty training stakeholders and identified seven inclusive learning climate strategies, of which three were prioritized for implementation in two training institutions.

**Supplementary Information:**

The online version contains supplementary material available at 10.1186/s12909-024-05521-z.

## Introduction

Following international migration trends [1, 2], diversity among students and trainees is growing [3, 4], with each of them bringing their specific cultural values, family- and migration histories [5]. However, postgraduate medical ethnic minority GP-trainees still face underrepresentation [3, 4] and may encounter unequal opportunities for success compared to their majority peers [6–9]. Learning climate-related factors, notably those related to lacking inclusiveness, likely play a pivotal role in this discrepancy [10–12].

Educational opportunities in GP-specialty training primarily rely on in-clinic learning, encompassing formal and informal contexts. Formal learning, characterized by structured, planned, and accredited activities within educational institutions, coexists with less structured informal learning, which is self-directed and arises from in-clinic everyday experiences and interactions, often susceptible to unspoken norms. While both approaches complement each other in providing a well-rounded education, the informal context might inadvertently reflect dominant cultural values and attitudes, potentially affecting in-classroom learning [13–15]. Particularly for ethnic minority GP-trainees, this lacking transparency may contribute to an increased risk of facing underperformance assessments, as these unspoken norms and values may not be self-evidently familiar to them [10–12].

Learning environments are subject to complex dynamics. Understanding the interconnected constructs of these dynamics is crucial for implementing transformative changes [16]. Accordingly, changes for inclusive learning opportunities require input from all organizational layers [17].

## Study objectives

With this study, we aimed to develop broadly supported recommendations for an inclusive learning climate in Dutch GP-specialty training.

## Methods

### Design

We used a qualitative Participatory Action Research (PAR) approach [18, 19], applying Participatory Learning and Action (PLA) techniques in stakeholder groups combined with insights from literature (Appendix) along with GP-trainees’ experiences related to inclusive education, to actively engage stakeholders in an inclusive dialogue [20–22]. This approach supported co-ownership, promoted compatibility with the organization’s actual needs, and facilitated successful implementation [23].

We employed two conceptual frameworks to shape the topic lists for stakeholder groups and guide result analysis.

**Fig. 1:**
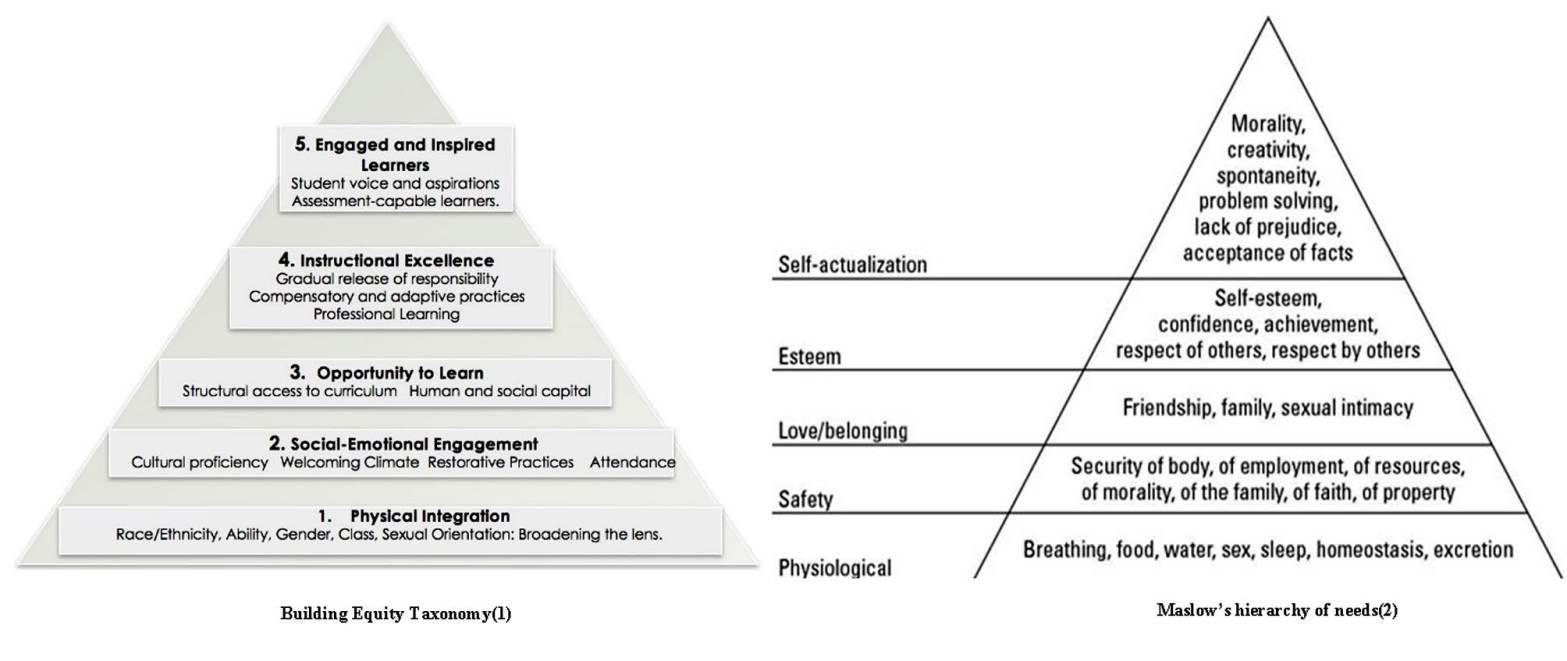
Building Equity Taxonomy [24] compared to Maslow’s hierarchy of needs [25]

**Fig. 2:**
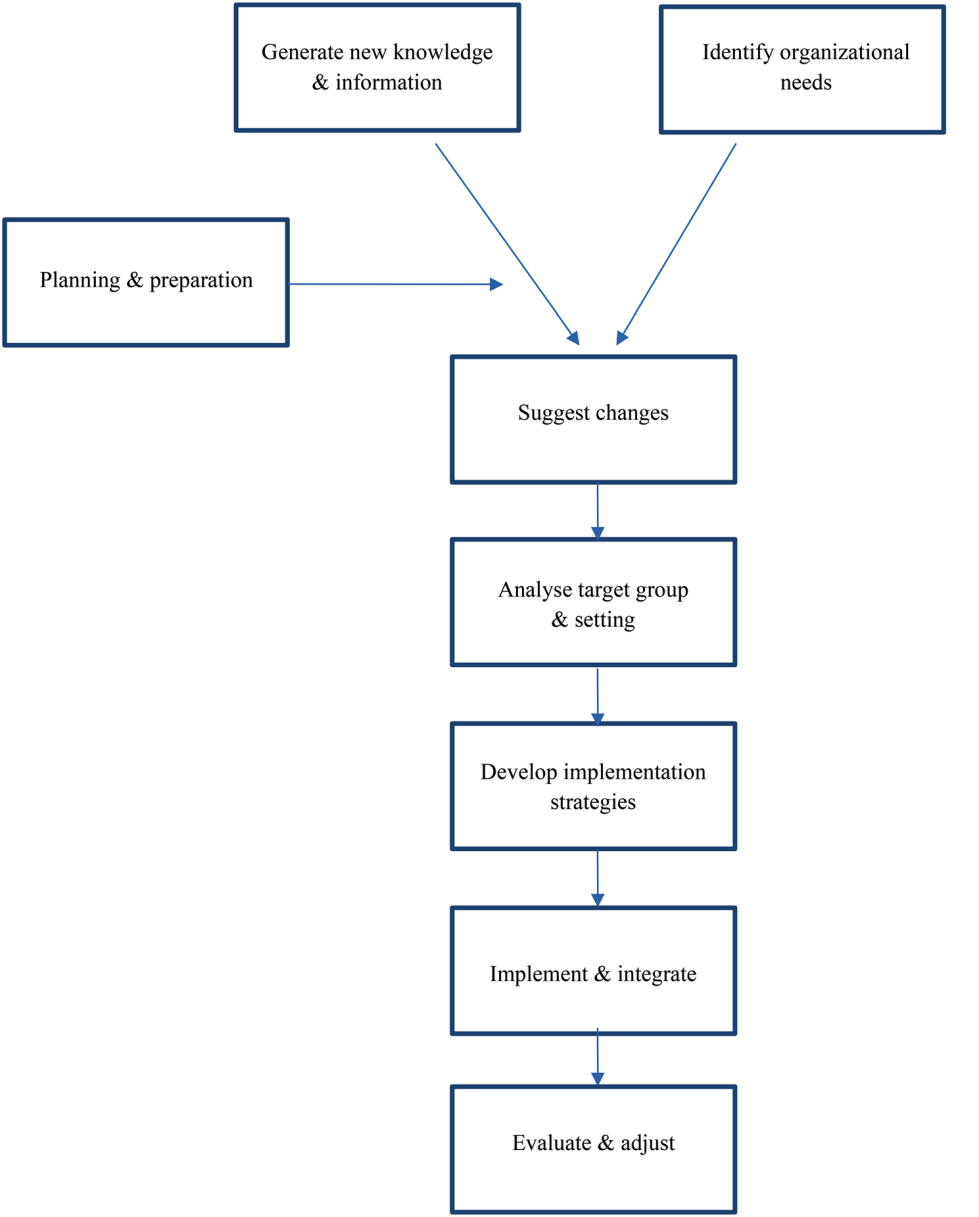
The Wensing & Groll model for implementation guidance [30]

1. The Building Equity Taxonomy (BET) framework for Diversity, Equity, and Inclusion (DEI), addressing students’ needs for equal educational opportunities, and covering the areas of physical integration, social-emotional engagement, equal learning opportunities, instructional excellence, and fostering inspired learners [24, 25] (Fig. 1). This framework is relevant to various educational settings, including GP-specialty training [12, 26–29].

2. The Wensing & Grol framework implementation guidance, equivalent to the internationally recognized Consolidated Framework for Implementation Research (CFIR) [30]. It provides implementation guidance for complex organizations, including clinical healthcare and educational settings [30, 31] (Fig. 2). This framework underpinned our implementation guidance, which the management team used for prioritization.

### Setting

This study took place at Amsterdam UMC’s two GP-specialty training institutes (AMC and VUmc). These institutes have demonstrated commitment to inclusiveness in their 2020–2022 annual reports, and they collaborate with the six other Dutch GP-specialty training institutes under GP-specialty Training Netherlands (HN).

One in three medical graduates in the Netherlands aims to enter GP-specialty training. In response to national medical demands, HN annually expands its acceptance of new trainees, projecting 921 in 2023 and an anticipated 1,190 in 2024, distributed across eight training institutes. About 17% of these trainees belong to ethnic minority groups, with most having completed pre-training at Dutch Medical Schools and a smaller group having graduated abroad [7]. Due to General Data Protection Regulation (GDPR) restrictions, the precise distribution of minority trainees across the eight national institutes remains undisclosed. However, a prior quantitative study indicated that by 2023, our pilot institutes showed a relatively proportional representation of Dutch GP-specialty training [7]. However, it is essential to note that qualitative research emphasizes a thorough description of the setting to enrich readers’ contextual understanding rather than strict representativeness.

The Dutch GP-specialty training program is a three-year dual-track program, supporting professional growth by combining in-clinic experience learning with one-day-a-week in-faculty education. Entry assessments aspire to guarantee the applicants’ knowledge, motivation, and Dutch proficiency. The program includes protocolled assessments, such as practical observations, systematic testing, and reviews of learning objectives.

### Study population

**Fig. 3:**
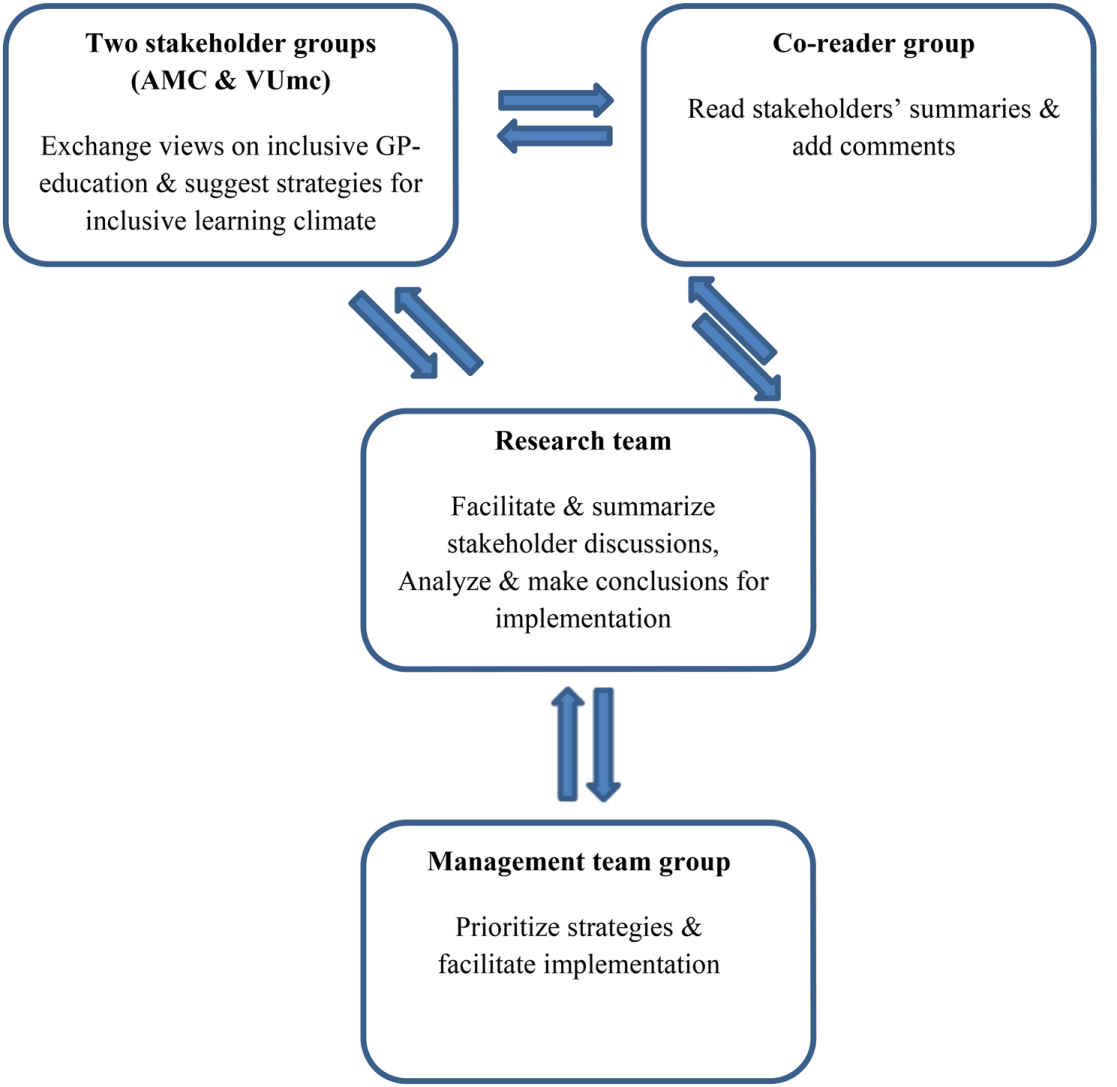
Participant groups

Acknowledging the essential need of broad support for inclusive organizational changes, we engaged participants from all backgrounds represented within the organization. Our study population encompassed the ethnic majority background as well as diverse ethnic backgrounds across all organizational layers (ranging from support personnel, management, educational staff (comprising both faculty and clinical educators), and trainees themselves), divided into two stakeholder groups, one co-reader group, and a management team group (Fig. 3).

Aiming to prevent eligible participants from experiencing researchers’ pressure, researchers sent information letters to team leaders, requesting them to forward in-faculty teachers, in-clinic supervisors, supporting bureau and management personnel, and trainees. From those interested, we purposefully selected twelve participants (six in each stakeholder group), striving for diversity regarding the position in the institute, age, gender, and ethnicity [32]. Stakeholders ranged from supporting bureau and management personnel (further in this text referred to as ‘staff’) to trainees, in-faculty teachers, and in-clinic supervisors representing diverse minority backgrounds as well as the majority background.

Stakeholder groups, each representing one GP-specialty training institute, provided input for inclusive strategies. Additionally, a co-reader group comprising interested individuals not in the stakeholder groups provided further insights through written comments. These groups represented diverse organizational layers, cultural backgrounds, ages, and gender. Representatives from management teams then evaluated and prioritized the suggested strategies.

### Data collection

**Fig. 4:**
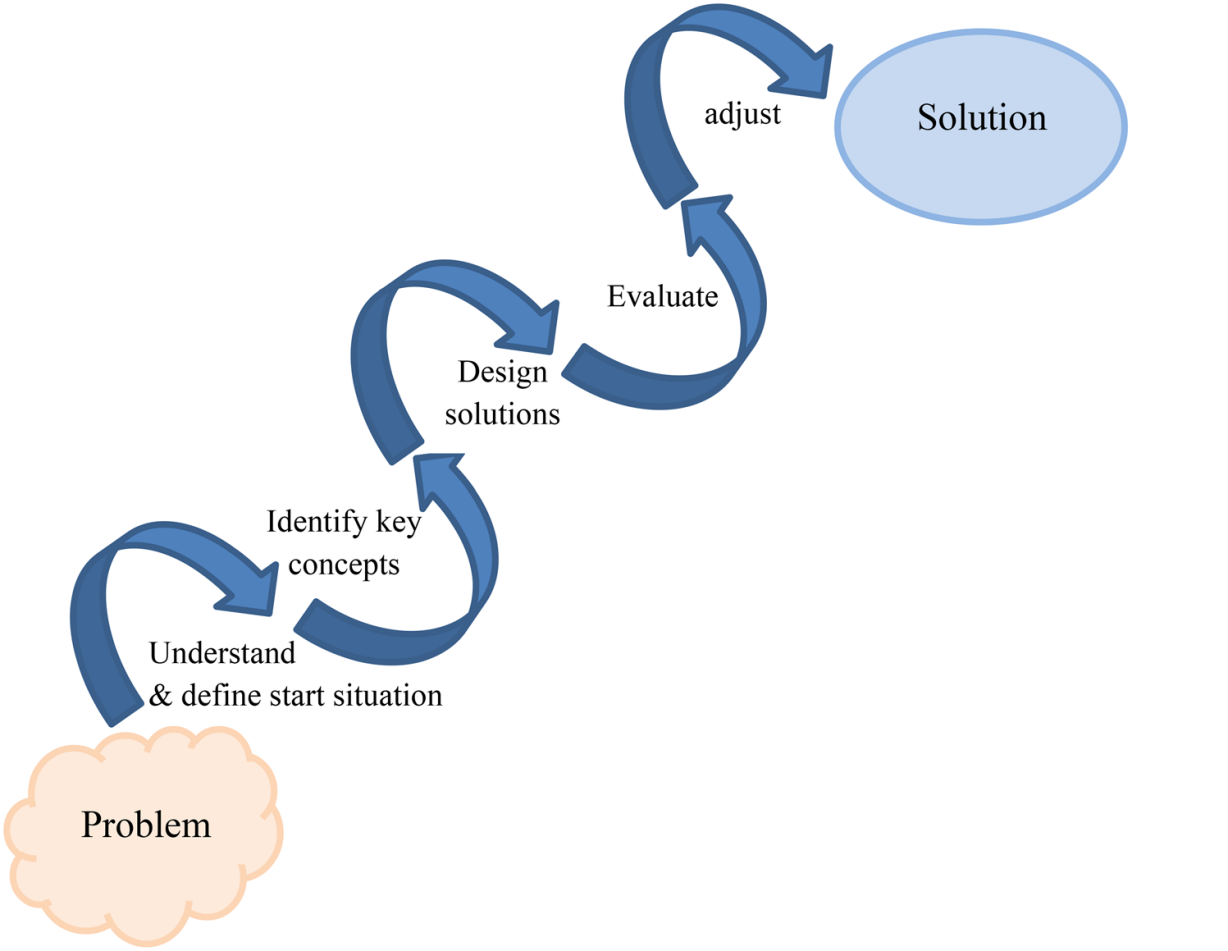
Cyclic phases until consensus of stakeholder groups’ processes [33]

Data collection and analysis took place from January 2021 to December 2022. Two researchers (MN, NvM) familiar with PLA-techniques facilitated six 90-minute PLA-based sessions for each stakeholder group. The sessions focused on inclusive learning environments and GP-specialty training’s inclusivity. In a cyclical process [33](Fig. 4), participants engaged in PLA techniques such as ice-breaking, flexible brainstorming, free-associating, direct ranking, mind-mapping, and visual evaluation. These methods facilitated sharing experiences and opinions and aligning these with relevant literature (Appendix, Table 1) to identify suitable inclusive strategies. After each stakeholder group session, the facilitator-researchers held debriefing sessions to reflect on their roles and identify areas for improvement. Independently, they summarized the key findings from each session and reached consensus through discussions. They presented these summaries in subsequent sessions for a member check and made adjustments based on participants’ feedback. To ensure a broader perspective, the co-reader group commented anonymously on these approved summaries, allowing them to contribute their personal perspectives, opinions, and experiences freely. Stakeholder groups then discussed and implemented these comments in their final session (Fig. 3).

**Table 1:** Characteristics of stakeholder-, co-reader-, and management group participants

	Stakeholder group 1	Stakeholder group 2	Co-reader group	Management team group
Willingness to participate	6 willing to participate	6 willing to participate	16 willing to participate	6 willing to participate
Withdrawal	-	-	3 withdrew because of time constraints	-
Age range	24–51 years old	23–53 years old	24–54 years old	45–60 years old
Gender	1 males 5 females	3 males 3 females	3 males 10 females	1 male 5 females
Position in GP-specialty training	1 in-faculty teacher 1 in-clinic supervisor 2 staff members 2 GP-trainees	1 in-faculty teacher 1 in-clinic supervisor 1 staff member 3 GP-trainees	6 in-faculty teachers 4 in-clinic supervisors 1 staff member 2 GP-trainees	2 Department Heads 1 Deputy Head 1 educational expert 2 curriculum coordinators
Ethnicity	3 ethnic minority 3 majority	3 ethnic minority 3 majority	6 ethnic minority 7 majority	0 ethnic minority 6 majority
Total	6 participated	6 participated	13 participated	6 participated

The stakeholder group topic list focused on:

1. Exploring :
  - The initial educational context;
  - Potential learning climate-related disparities;
  - Out-of-the-box wishes and key elements for an inclusive learning climate;
2. Strategy developing and preparing for implementation:
  - Recommendations for inclusive GP-specialty training;
  - Mapping onto the BET framework’s hierarchical levels of DEI [24](Fig. 1);
  - Translating recommendations into actionable strategies.
  - Identifying Wensing & Grol conditions and requirements for implementation [30](Fig. 2).

Due to the Covid-19 pandemic, we adapted the study’s in-person design to online methods for creative brainstorming. In these virtual sessions, physical distance and potential distractions of personal environments challenged trust and commitment, especially for GP-trainees who felt vulnerable sharing ideas with in-faculty teachers, in-clinic supervisors, and staff, who might also be their assessors in daily educational contexts [34]. To address this risk, we dedicated extra time, and utilized online tools: Zoom 5.13.11 for breakout rooms, Padlet 200.0.0 for visualizing PLA techniques, and concise PowerPoint presentations for member check summaries and goal-setting [35].

The facilitator-researchers (NvM, MN) collected audio recordings and written co-reader comments. An external bureau transcribed audio-recordings verbatim.

One researcher (NvM) regularly presented our findings during periodic staff meetings. These presentations not only aimed to keep the entire team informed but also played a crucial role in garnering broader support and incorporating diverse opinions for our project.

### Data analysis

Within three days after each session, we (NvM, MN, and MV) analyzed the transcribed audio recordings and written co-readers’ comments, and discussed our analyses until consensus.

To provide actionable qualitative insights while responding to ongoing participant feedback, we adopted an inductive rapid qualitative data analysis approach inspired by Hamilton’s model [36–39]. This method prioritizes identifying key elements and mechanisms over extensive theoretical insights. Through structured data collection using topic lists and Participatory Learning and Action (PLA) techniques, along with expedited transcription, we efficiently analyzed ideas and condensed findings into concise formats like post-interview notes and matrix summaries. Although not a traditional thematic or framework analysis, we employed theme-informed and framework-informed codes to organize data, considering context and group dynamics, which allowed us to explore interactional group dynamics and communication styles in the participants’ discourse and its points of consensus or contention within specific statements [40]. We anticipated this method, aligned with the literature, to yield qualitative outcomes as consistent and rich as traditional in-depth transcription coding while facilitating the analysis of interconnected sessions [36, 41, 42].

We analyzed the stakeholders’ ideas, recommendations, and their identifyed Wensing & Grol conditions and requirements for implementation to create implementation guidance [30]. This guidance encompassed analyzing organizational structure, identifying change potential and barriers, defining the target population, describing tailored DEI-strategies, estimating timelines for internalization processes and implementation, and designing evaluation methods (Fig. 2). Subsequently, we invited management group participants for hybrid (online and in-person) meetings, where they engaged in substantive discussions to evaluate this guidance and prioritize recommended strategies, based on the expected feasibility and compatibility with their setting.

### Reflexivity and ethics

Two authors, NM and MN, identify as minority females. While their unique backgrounds enhance sensitivity towards minority peers’ experiences, a potential challenge arises where these experiences resonating with them might be more salient. To mitigate this, we organized reflective debriefing sessions addressing diverse viewpoints and emphasizing the researchers’ roles as instruments in data collection and analysis. During these sessions, we engaged in candid discussions probing our experiences, expectations, preoccupations, and opinions that could have influenced our approach to data collection and analysis.

Also, the roles of participating stakeholders may have influenced views they shared in this research process. They spanned all organizational positions, ranging from department heads to trainees, in-faculty teachers, and in-clinic supervisors, representing both, majority and minority backgrounds. While deliberately seeking these varied insights, we remained mindful of potential power dynamics influenced by different positions or ethnic backgrounds. To foster a safe space and address these dynamics, facilitators employed PLA-techniques, such as ice-breakers. Also, they established clear agreements with all stakeholder group members regarding privacy, openness to differing views, and ensuring safety. Should any commitments be breached, facilitators were trained to address them promptly. In fact, stakeholders demonstrated remarkable respect and curiosity towards understanding each other’s perspectives throughout the process.

## Results

### Participant characteristics

Table 1 presents participant characteristics for the stakeholder, co-reader, and management groups. In total, 31 stakeholders participated, aged 24 to 60, including eight males, 24 staff members from diverse organizational positions, seven trainees, and 12 ethnic minority participants.

### Attendance

The stakeholder group sessions had an attendance rate of 97%. All co-readers responded to the request for comments. During the hybrid management group session, 40% of participants attended in-person, while 60% joined online.

### Stakeholder group sessions

In line with the topic list, we organized the results into two sections: [1]Exploring and [2] Strategy developing and preparing for implementation. In Sect. 2, the stakeholders aligned their results with the BET framework and structured them according to the Wensing & Grol framework.

#### Exploring

##### The initial educational context

Stakeholders defined inclusiveness in the GP-specialty training as collective curiosity and support for trainees’ unique professional identities, regardless of their characteristics or backgrounds. As preconditions for in-faculty teachers, in-clinic supervisors, and staff, participants mentioned [1] willingness to encounter emotional discomfort [2], embracing failures in order to learn, and [3] acknowledgement of unconscious bias.

*‘… we will not always succeed to be without prejudice, that is allowed as long as we will put the effort in gaining awareness’* (participant 2, group 1).

Participants emphasized creating a safe learning environment where all voices, including minority voices, can be heard. They suggested reflective questions starting with:

‘*Could you imagine that…’.*

Participants highlighted parallel processes whereby educators foster trainees’ personal and professional development, and GPs support patients’ individual coping styles. Such an inclusive and safe learning environment would act as a flywheel, enhancing the institute’s inclusive image and attracting prospective minority trainees, teachers, and in-clinic supervisors.

Co-readers confirmed these view points and they added their concerns regarding prioritization by some staff members:

*‘I have nothing to add. I think it is essential that diversity is given a priority, that we as staff all agree that this is important. The pitfall is that some of them might not see the importance’.* (co-reader 2)

##### Potential learning climate-related disparities

Stakeholders from ethnic minority groups expressed distress experiences in a dominant white world:

*‘The GP-specialty training population is predominantly white and female; trainees, in-faculty teachers, and in-clinic supervisors even seem to resemble one another. Without them saying or acting, I continuously feel the stress of having to adapt to them, which I will never be able to’* (participant 2, group 1).

Stakeholders discussed the majority’s naivety in understanding the experience of belonging to a minority and expressed concerns about some DEI programs potentially leading to paradoxical stigmatization. They noted instances where in-faculty teachers appointed minority trainees as representatives for their cultural groups, ignoring the vast diversity within these groups. Also, participants reported stereotyping case reports:

*‘They always use the example of the non-Dutch speaking overweight Moroccan mother of seven children, not engaged in any sports, who favors sweet and fatty food, and suffers from diabetes’* (participant 3, group 2).

Co-readers added that this one-sided picture made minority trainees uneasy, feeling discussed rather than equal partners in GP-training. Additionally, they emphasized that presenting DEI programs as non-mandatory, implied that diversity and inclusiveness were not necessarily integral to GP-skills requirements.

*‘Mandatory inclusive training for mentors, staff, and teachers holds significant importance, signifying our commitment. Participation in these courses should be integrated into evaluations and annual interviews’.* (co-reader 4)

##### Out-of-the-box wishes and key elements for an inclusive learning climate

Upon the invitation to make a wish:

*‘Wouldn’t it be wonderful if….‘*,

stakeholders wished for diverse staff as role models, willing to learn from each other, normalizing various meaningful insights, and embracing diverse worldviews:

*‘By using these differences, we keep each other awake and open-minded in exploring possibilities; thus, we allow ourselves to grow without assuming that our paved path is always the best way at the time’* (participant 3, group 1).

Stakeholders indicated the institute’s responsibility to educate GP-trainees for a diverse patient population as an essential component of an inclusive learning environment. Key elements related to such inclusiveness were:

The GP-specialty training should represent society in all its diversity:

*‘It’s been a few years since I started GP-specialty training, of course, but… I’m just digging whether I had a feeling of: “I fit in there” or: “I recognize my roots there”. These are important feelings to me to feel safe at my work- and study place’* (participant 7, group 1);

A diverse GP workforce meets patients’ appreciation for GPs they can identify with:

*‘Regarding this cultural background or ethnicity, I have the impression that patients from ethnic minorities often liked that I obviously am not Dutch, they said, “oh, you are not Dutch, are you?“, it led to recognition, a little laugh, and connected us. Having a doctor just like them helped my patients to share their concerns.’* (participant 2, group 2);

GP-trainees need identifiable and diverse educational role models:

*‘The moment you sit down together and see that diversity, …brings different working styles, learning styles, or communication styles… that you realize we have to do it together, the greater the diversity, the more we learn from one another, the higher we rise, the more fun and creative ideas…’* (participant 4, group 2);

Diverse GP-trainee cohorts improve mutual learning processes:

*‘To me, utilizing diversity means that there’s always someone in the classroom who says, “Okay, so what if we look at it from that perspective or through those glasses?’* (participant 1, group 1).

Co-readers agreed and added that GP-specialty training already utilized diversity among in-faculty teachers to some extent:

*‘Great idea! Diversity among teachers is already being leveraged to some extent. Trainees can synthesize a blend of styles and insights from different teachers and mentors. Expanding on this concept could help cultivate a more inclusive learning environment’*. (co-reader 1)

#### Strategy developing and preparing for implementation

##### Recommendations for an inclusive GP-specialty training

Participants (stakeholders in collaboration with co-readers) made six fundamental recommendations and mapped these onto the BET framework levels to ensure all aspects of inclusive education would be covered [24] (Table 2).

**Table 2:** Stakeholders’ and co-readers’ recommendations related to Building Equity Taxonomy (BET) levels

Stakeholders’ and co-readers’ recommendations		BET levels
a.	Create support in all layers of the organization	1–2
b.	Express core-values clearly to all stakeholders	1–2
c.	Create Diversity, Equity, and Inclusion (DEI)-knowledge networks	3–4–5
d.	Support a solid and inclusive learning community	2–3–4–5
e.	Support self-actualization for minority trainees	1–2–3–4–5
f.	Provide tailored, mandatory DEI-training to teachers, staff, and trainees	1–2–3–4–5

##### Actionable strategies

From these recommendations, participants derived seven actionable strategies for promoting inclusive GP-specialty training (Table 3).

**Table 3:** Suggested actionable strategies

Strategies	Aims	Actions	Target audiences	Alignment with recommendations & Building Equity Taxonomy (BET) levels (as displayed in Table 2)
Provide a clear message of inclusiveness in all internal and external communication	Promote Diversity, Equity, and Inclusion (DEI) mission & vision Attract applicants who identify with DEI mission & vision	Revise & edit existing ‘business cards’ Revise & edit educational material and language Utilize (digital) platforms for target groups Consult expert DEI- & communication advisors	(Aspiring) trainees, staff, teachers, trainers, etc.	Recommendations: b - d - e BET levels: 1–2–3–4–5
Appoint DEI ambassadors in all layers of the organization	Durable support for the DEI mission and vision Attention for DEI issues in all teams Sense of belonging for all individuals involved Profound knowledge networks	Define ambassador job description Invite staff members and trainees to apply Provide relevant professionalizing training programs	Trainees, staff, teachers, trainers	Recommendations: a - c - d - e BET levels: 1–2–3–4–5
Facilitate procedures for secure incident reporting	Support against exclusion and (micro)aggression experiences Trend identification Normalized DEI reflection within teams & clear improvement goals	Design accessible reporting procedures Create, adapt and evaluate reporting questionnaires Create structure for addressing individual reports, discussing trends, and setting improvement goals Appoint DEI confidants Provide professionalizing trainings	Trainees, staff, teachers, trainers	Recommendations: a - b - d - e BET levels: 1–2–3–4–5
Give a significant voice to minority trainees in ongoing program development	Normalization of a diverse voice Ownership for minority trainees Optimal learning benefits of the in-group expertise Lessons in listening and reflecting for majority colleagues	Attract minority trainees, teachers, and staff Invite minority trainees for round-table discussions	Trainees, staff, teachers, trainers	Recommendations: a - b - c - d - e - f BET levels: 1–2–3–4–5
Assign multiple teachers and mentors as diverse role models	Multiple identifiable role models for trainees with inherent increased recognizibility Empowerment of underrepresented minority trainees	Two teachers per in-faculty group Dual GP-trainers per in-clinic learning place	Trainees, teachers, trainers	Recommendations: a - d - e BET levels: 1–2–3–4–5
Offer just-in-time learning	Societal engagement Learning from the available in-group expertise Knowledge, skills and attitudes for healthcare in a diverse society	Create knowledge exchange platform for societal relevant topics Create an online knowledge platform for just-in-time DEI education Equip teachers and GP-trainers for just-in-time education	Trainees, teachers, trainers	Recommendations: d - e - f BET levels: 1–2–3–4–5
Provide mandatory DEI-relevant training programs for professional development	DEI awareness Improved DEI communication skills and leadership qualities Professional growth through reflection and exchange Strengthened educational community	Identify DEI learning needs through interviews and questionnaires Search and/or develop training courses accordingly Evaluate and adjust	Trainees, staff, teachers, trainers	Recommendations: a - b - c - d - e - f BET levels: 1–2–3–4–5

1. *Provide a clear message of inclusiveness in all internal and external communications*.

Participants explored various means and media platforms for promoting the GP-specialty training’s DEI core values (websites, ads, social media, podcasts), focusing on design, content, and appeal to the target group. They recommended involving trainees with media experience rather than exclusively hiring specialized communication consultants.

2. *Appoint DEI ambassadors in all layers of the organization*.

Participants suggested involving employees as DEI ambassadors to effectively spread DEI core values in the organization. Ambassadors would undergo comprehensive training in DEI, reflective skills, leadership, and change management. They would also attend conferences, masterclasses, join knowledge networks, and contribute to think tank initiatives as part of their preparation.

3. *Facilitate procedures for secure incident reporting*.

Participants highlighting the significant impact of unintentional discriminatory behavior, often resulting in experiencing barriers to reporting such incidents. They proposed implementing low-threshold and secure reporting procedures with targeted questions on DEI and (micro)aggression. Regular team sessions would enable open discussions based on anonymous reports, fostering inclusive education, uncovering organizational trends, and providing support for trainees who faced discrimination, microaggression, or exclusion. Confidential advisors would receive training in DEI, reflective skills, and relevant legislation.

4. *Give a significant voice to minority trainees in ongoing program development*.

Participants advised inviting minority trainees to round table discussions, fostering insider perspective exchange with mutual respect, critical reflection, and empathy. Including these diverse voices would promote resilience and professional growth and attract eligible trainees and staff from diverse backgrounds.

5. *Assign more than one in-faculty teacher per group / in-clinic training*.

GP-trainees - like all individuals - naturally mirror the behavior of significant others, such as teachers, in-clinic supervisors, or peers. Participants believed that trainees with multiple role models would outperform those with single role models. They suggested introducing dual in-faculty teachers and dual in-clinic supervisors as additional role models and an extra pair of eyes during education. To ensure success, participants recommended training programs for optimum role model utilization.

6. *Offer ‘just-in-time’ learning*.

Participants agreed that effective learning is closely related to immediate learning needs. For GP-trainees, such learning needs often arise from societal encounters in the consultation room, e.g., guiding Muslims during Ramadan while simultaneously managing diabetes or comprehending increasing PTSD symptoms around Keti Koti (Afro-Surinamese Emancipation Day). Timely incorporating these contextual factors into training programs could provide directly applicable knowledge.

7. *Provide mandatory DEI relevant training programs for professional development*.

Participants emphasized the necessity of new knowledge, skills, and attitudes. They considered within-group differences valuable learning tools for diverse personal and professional development paths. Well-trained staff and trainees could drive inclusive knowledge networks, empower the organization, and positively influence external perceptions. Thus, they recommended mandatory and tailored training programs aligned with the anticipated learning needs from the suggested strategies. Where applicable, they advised considering outsourcing.

##### Conditions and requirements for implementation

Participants indicated the importance of in-faculty teachers, in-clinic supervisors, and staff having the courage to be vulnerable. They emphasized the essence of transparent norms and values and a welcoming learning environment, and they highlighted an attitude of:

*‘… genuinely enjoying to support a diverse population in their growth towards their professional identities’* (participant 6, group 2).

*‘Implementing these ideas demands courage and vulnerability, particularly as their execution could inadvertently carry stigmatizing effects’.* (co-reader 6)

In this context, they mentioned the risk of unconscious bias, which could require external expert trainers at certain stages:

*‘Well, you know, I had a trainee of Moroccan descent, and it shocked me that, while I always thought to be very open, diversity-minded, and curious for everything and everyone, I found it way more difficult to connect than I’d admit. I wonder what would have helped me unveil this blind spot in an earlier stage…’* (participant 5, group 1).

*‘… allow and embrace the differences, see them as opportunities that actually add learning qualities, and not take them away? So, professionalism will become more colorful, and it can be viewed from different points of view, not just the traditional, established perspectives and routes’* (participant 1, group 1).

Ultimately, we provided the management group with implementation guidance for these seven strategies, along with an analysis of the target group and context, and summaries of relevant literature on DEI best practices in educational settings (Appendix). The management team agreed that enhancing DEI should have priority in Dutch GP-specialty training:

*‘We should acknowledge that we are trailing behind societal advancements in diversity. Therefore, maintaining a strong focus on this topic must stay a priority’* (participant 5, management group).

Based on these comprehensive data, the management group prioritized strategies that covered the overarching recommendations and BET-levels (detailed in Table 3; Fig. 1), which aided in selecting strategies with anticipated effectiveness. To enhance alignment with the organizational requirements and feasibility, they considered implementation requirements, staff feedback from our presentations during periodic meetings, and opportunities for synergy with existing projects in other Amsterdam UMC departments.

*‘We can see that literature describes these strategies as effective and we assume that stakeholders meticulously aligned them with the institute’s needs. Let us not repeat that process but rather look into strategies that can be implemented effectively in our setting’* (participant 1, management group).

*‘For each suggested strategy, this guidance envisions its coverage and practical implications. Now, it is up to us to consider how far we are willing to commit. This process prompts pertinent questions on specific effective actions’* (participant 2, management group).

The management group prioritized three strategies:

- Appoint DEI ambassadors in all organizational levels,
- Give a significant voice to minority trainees in ongoing program development,
- Provide mandatory DEI-relevant training programs for professional development to all involved in GP-specialty training.

## Discussion

### Summary of findings

In twelve PLA-based stakeholder sessions, participants explored perspectives on potential disparities, underlying causes, and aspirations for an inclusive learning climate in the Dutch GP-specialty training. They suggested seven strategies based on six overarching recommendations, which they presented embedded in an implementation guidance to the management group:

1. Provide a clear message of inclusiveness in all internal and external communications.
2. Appoint DEI ambassadors^1^ in all layers of the organization
3. Facilitate procedures for secure incident reporting.
4. Give a significant voice to minority trainees in ongoing program development.
5. Assign more than one in-faculty teacher per group / in-clinic supervisor per trainee.
6. Offer ‘just-in-time’ learning.
7. Provide mandatory DEI relevant training programs for professional development.

The management team selected strategies 2, 4, and 7, deeming them most effective, feasible, and aligned with the organization’s requirements.

### Comparison to existing literature

Worldwide attention to inclusive learning climates in postgraduate medical education revealed the complexity and multidimensionality of educational constructs and institutes [29, 43]. Interpretations of formal and informal learning contexts within these environments depend on the perspectives of various stakeholders [15]. Consequently, unconsciously normalized rules and codes across all layers may implicitly exclude ethnic minority professionals and -trainees in many ways throughout their careers [44].

This paper extends the literature on inclusive GP-specialty training [15, 43, 44], detailing the efforts to design- and create broad support for inclusive training strategies. Like most organizational changes, implementing inclusive strategies in GP-specialty training posed challenges and demanded a focus on building confidence and trust in novel approaches [45]. Hence, understanding the values and expectations of target groups and tailoring strategies to meet their needs and aspirations was crucial. Our study involved representatives from all key stakeholders, including ethnic minority trainees, aiming to address critical research gaps and enhance knowledge quality, relevance, and impact [46]. Collaborative decisions, rooted in an equal and reciprocal partnership, empowered stakeholders, raised management team awareness and inspired the research team [47]. These effects mirror findings in previous PAR studies on inclusive primary healthcare [48] and highlight PAR’s role as a catalyst for transformative change in GP-specialty training [33].

Stakeholder insights, combined with DEI-strategy literature, underscored the need for a gradual, committed cultural shift towards inclusivity in the learning environment. Based on these insights, the management group recognized that this transformation would necessitate a set of strategies addressing inclusiveness at various levels rather than relying on one single intervention [26, 28, 49, 50]. They employed our Wensing and Grol-based implementation guidance to select the following feasible strategies aligned with the GP-specialty training context as a first step in an ongoing process:

- *Providing mandatory DEI-relevant training programs to all stakeholders* supports cultural responsiveness within all strategies to be implemented. It facilitates understanding how cultural backgrounds and experiences influence teaching and learning [49]. Ultimately, it fosters engagement and motivation to create collaborative learning environments and accommodate learners’ needs based on their diverse backgrounds [26].
- *Appointing DEI ambassadors in all layers of the organization* has in other contexts proven to enhance the effectiveness of DEI-related strategic initiatives [51]. DEI ambassadors engage change agents within their teams, foster collaboration and effective communication, facilitate diversity goals, and involve key stakeholders in sustainable, inclusive changes [50].
- *Giving a significant voice to minority trainees* empowers and amplifies their agency. Including their experiences and perspectives in staff meetings and brainstorming sessions is a crucial first step toward an open and innovative culture. Prior research indicated that promoting minority trainees’ participation requires supportive supervision, encouraging them to share transformative ideas [28].

### Strengths and limitations

Our participatory approach fostered broad support across all organizational levels. PLA-based stakeholder discussions facilitated open dialogue, refined ideas, and sparked valuable insights. Co-reader feedback prompted stakeholder group participants to reevaluate their interpretation of specific experiences. This approach allowed diverse perspectives and theoretical idea saturation, aiding participants in identifying seven actionable strategies with high potential for effective implementation. In turn, these results allowed the management group to leverage their organizational expertise and prioritize three strategies they considered feasible and compatible with the organization’s requirements.

While most post-graduate medical education settings share similarities, contextual variations, such as educational emphasis and cultural factors, may exist, leading to potential limitations in the transferability of our findings. Nonetheless, the dynamics between informal and formal in-classroom learning remain pertinent across various postgraduate medical contexts, where in-clinic learning, shaped by day-to-day experiences and supervisor-trainee dynamics, inevitably influences formal learning objectives and settings. Also, our study’s confinement to two Dutch GP-specialty training institutes and its relatively modest participant count may require caution in the transferability of our findings to other similar settings. In light of this, it is noteworthy that statistics from a previous quantitative study suggest that by 2023, our pilot institutes closely mirrored Dutch GP-specialty training in terms of minority trainee [7]. Moreover, we provided meticulous descriptions of our setting to enhance contextual understanding, aiding in assessing transferability to similar settings. Additionally, the explicit commitment to inclusiveness by the participating GP-specialty training institutions, which could be instrumental in promoting successful implementation, could pose challenges when transferring the results to less DEI-focused settings.

Still, employing multiple sources by connecting stakeholder perspectives to relevant literature and two frameworks enabled participants to structure their thoughts and opinions on the organization’s DEI strengths and limitations, along with the opportunities and challenges for implementation. For future researchers, this approach may prove valuable in identifying overarching concepts and theories that transcend specific individuals or contexts and facilitate the assessment of the transferability of our findings to similar educational settings [52–55].

### Implications for further research and practice

Fostering a DEI-minded culture in post-graduate medical training calls for a multifaceted strategy. As training institutes diversify and curricula address nuanced topics, skills for adeptly navigating complex conversations become increasingly critical for educational staff. The ongoing process of promoting inclusive teaching, assessment, and curriculum design abilities will necessitate the inclusion of a wide range of perspectives. Consequently, we recommend involving stakeholders from the most diverse backgrounds possible. Also, the explicit commitment to inclusiveness by the participating GP-specialty training institutions may pose challenges when transferring the results to less DEI-focused settings. Therefore, we suggest further investigation in such contexts to better understand the transferability of our results.

Ensuring high-quality, inclusive learning environments in postgraduate medical education is crucial for educational opportunities and the overall quality of healthcare [56]. However, this inclusiveness is not solely shaped by the beliefs and values of teachers; it is also intricately influenced by the complex social and cultural dynamics within educational institutions [29]. Inclusiveness strategies are catalysts for enduring cultural transformation, demanding the consistent integration of multiple strategies through incremental steps over an extended period [43]. The three strategies identified in our study, which were prioritized for implementation, represent initial strides toward instigating this cultural transformation. Subsequent phases involving evaluation, adaptation, and implementation of additional strategies are imperative for sustaining engagement in a culture of inclusive postgraduate medical education. All Dutch GP-specialty training institutes closely monitor our findings and have committed to implementing mandatory DEI-relevant training programs for their staff and trainees.

Additional research on the impact of the implemented strategies and the level of stakeholder engagement throughout the implementation phase is needed. This follow-up research should encompass inclusive teaching methods, assessment strategies, curriculum design, attitudes, and the ethnic minority trainees’ experienced inclusion aligned with the BET framework.

## Conclusion

Engaging stakeholders in PLA-based sessions at two Dutch GP-specialty training institutes proved instrumental in identifying recommendations for an inclusive learning climate. Stakeholders identified seven tangible DEI-strategies, addressing all five BET aspects:

1. Provide a clear message of inclusiveness in all internal and external communications: enhances inclusive accessibility and a diverse learning community;
2. Appoint DEI ambassadors in all layers of the organization: promotes knowledge exchange, reflection on potential biases, and active engagement in DEI networks;
3. Facilitate secure DEI-incident reporting procedures;
4. Give a significant voice to minority trainees in ongoing program development: empowers them and creates reciprocal learning;
5. Assign more than one teacher per group / in-clinic training: creates multiple role models and perspectives;
6. Offer ‘just-in-time’ learning: fosters social and educational engagement;
7. Provide mandatory DEI-relevant training programs for professional development: promotes DEI-expertise and awareness among all involved.

Based on anticipated feasibility and effectiveness, the management group prioritized strategy numbers 2, 4, and 7 for implementation.

Our integrative approach supported collaborative, context-specific strategy development and prioritization, effectively balancing anticipated effectiveness and compatibility. As such, this approach will prove valuable in identifying widely supported DEI strategies within varying and complex post-graduate medical educational contexts.

### Electronic supplementary material

Below is the link to the electronic supplementary material.

Supplementary Material 1

## Data Availability

The datasets used and/or analysed during the current study available from the corresponding author on reasonable request.

## Footnotes

1 Ambassadors actively promote DEI values within teams by exchanging knowledge and experiences, enhancing expertise through training and literature, and addressing diversity cases through consultancy roles.
