# Supplementary material for: Towards inclusive learning environments in post-graduate medical education: stakeholder-driven strategies in Dutch GP-specialty training
Source: BMC Med Educ. 2024 May 17;24:550. doi: 10.1186/s12909-024-05521-z (PMC11100146; doi:10.1186/s12909-024-05521-z)
Supplement: Supplementary file 1 — Supplementary Material 1 [file 12909_2024_5521_MOESM1_ESM.docx]

**Appendix - Towards Inclusive Learning Environments in Postgraduate Medical Education: Stakeholder-Driven Strategies in Dutch GP-Specialty Training**

Table 1: Leveraging literature for stakeholder group sessions

| **Learning environment** | | |
| --- | --- | --- |
| **Publication** | **Conclusion** | **Relevancy for stakeholder discussions** |
| Boor, van der Vleuten et al., 2011 (1) | Learning climates are theoretical concepts that cannot be directly measured. Key factors influencing learning climates are the integration of work and training and the customization of experiences to individual residents' needs. D-RECT seems to be a valid, reliable, and practical tool for assessing these factors. | Understanding the complexity of (assessing) postgraduate medical learning climates |
| Trullas, Blay, et al., 2022 (2) | Problem-based learning is effective for medical education. Students will not only acquire knowledge but also other professional competencies. | Exploring the advantages of problem-based learning in medical education |
| Karp, Hauer, et al., 2019 (3) | Medical learners, including students and residents, rely on a foundation of trust in their educational setting. Educators and supervisors can foster trust by encouraging open discussions, which enrich the learning experience. | Understanding the trainees’ experienced key factors for safe learning climates |
| Cox, 1994 (4) | This book offers a comprehensive exploration of cultural diversity in organizations, addressing it on three levels (individual, group, and organizational) and from various angles (theory, research, and practice). | Understanding concepts of organizational inclusiveness |
| Carter and Darling Hammond, 2016 (5) | Diverse students with varied backgrounds and teachers with their own diverse experiences face challenges. Students' academic success depends on teachers' understanding of their lives, backgrounds, and learning needs, along with pedagogical knowledge. This diversity in schools leads to ongoing instructional challenges. | Exploring success factors for teaching in diverse settings |
| **Cultural awareness** | | |
| **Publication** | **Conclusion** | **Relevancy for stakeholder discussions** |
| Lee, Vaishnavi, et al., 2009 (6) | Demographic differences in student communication styles underscore the importance of cultural competency training for assessors. | Understanding the cultural influence on communication assessments in diverse trainee groups |
| Bullock, Lockspeiser, et al, 2020 (7) | Stereotype threat is common, particularly among non-White students, and interferes with learning. Increased minority representation and developing evidence-based strategies for allyship around microaggressions could mitigate effects of stereotype threat. | Understanding the threat of stereotyping for learning climates, and considering strategies to address this threat |
| Carter and Darling Hammond, 2016 (5) | Diverse students with varied backgrounds and teachers with their own diverse experiences face challenges. Students' academic success depends on teachers' understanding of their lives, backgrounds, and learning needs, along with pedagogical knowledge. This diversity in schools leads to ongoing instructional challenges. | Understanding learning needs of a diverse student population |
| Roberts, 2020 (8) | Belonging, feeling accepted and valued, significantly impacts health, relationships, professional success, and well-being. Discrimination, bias, and microaggressions disrupt this sense of belonging, leading to isolation, trust issues, emotional distress, reduced effectiveness, exhaustion, and health problems. Underrepresented groups are disproportionally at risk of experiencing these effects. | Exploring the importance of belonging |
| **DEI-strategies** | | |
| **Publication** | **Conclusion** | **Relevancy for stakeholder discussions** |
| Diaz, Navarro, et al., 2020 (9) | A diverse workforce is essential for addressing healthcare challenges. Achieving equity and inclusion requires institutional efforts like recruiting diverse faculty, teaching cultural humility, and fostering inclusivity. It's a responsibility shared by the entire medical community, demanding time, resources, and innovation. | Exploring required institutional DEI-efforts |
| Wusu, Tepperberg, et al., 2019 (10) | More minority physicians improve care access and quality. Family physician diversity lags behind the population. The authors developed a strategic plan: outreach to minority candidates, unbiased interview- and data analysis, increasing minority applicants and matched ones significantly. This plan can also benefit other institutions and specialties. |  |
| Cotton & Green, 2014 (11) | This chapter highlights new media as social capital for diversity officers. | Strategy development: *Provide a clear message of inclusiveness in all internal and external communications* |
| Kolluru, Wanat, et al., 2023 (12) | DEI committees are crucial for culture change to prepare graduates for equitable patient care. Effective DEI committees include passionate change agents, engaging the entire learning community for a shift towards inclusive organizational culture. | Strategy development: *Appoint DEI ambassadors in all layers of the organization* |
| Munn, 2017 (13) | Racist remarks from patients and colleagues are not uncommon. There is a lack of support and guidance for students on how to report incidents. | Strategy development: *Facilitate procedures for secure incident reporting* |
| Regenmortel, 2009 (14) | Empowerment involves connecting people, organizations, and groups, prioritizing vulnerable individuals, and using strengths-oriented care through appropriate methodologies within organizations and policies. | Strategy development: *Give a significant voice to minority trainees* |
| Rask & Bailey, 2002 (15) | Faculty members can serve as role models for women and minority students, influencing their choice of major when they share a similar background. | Strategy development: *Assign multiple teachers and mentors as diverse role models* |
| Haeger & Fresquez, 2016 (16) | Mentoring benefits diverse students academically. Those with mentors who offer socioemotional and culturally relevant guidance alongside professional mentorship show enhanced learning and development. Longer-lasting mentorships positively impacted student development. |  |
| Allis, 2020 (17) | Just-in-time learning concerns readily accessible, on-demand learning experiences. This paper provided examples and best practices for designing, developing, and implementing such learning experiences. | Strategy development: *Offer ‘just-in-time’ learning* |
| Bullock, Lockspeiser, et al, 2020 (7) | Stereotype threat is common, particularly among non-White students, and interferes with learning. Increased minority representation and developing evidence-based strategies for allyship around microaggressions could mitigate effects of stereotype threat. | Strategy development: *Provide DEI relevant training programs* |
| Lupton abd O’Sullivan, 2020 (18) | Bias affects underrepresented medical learners. Faculty are hesitant to address this issue due to fear of mistakes and limited training opportunities. The authors designed highly-rated DEI-skills workshops for teaching, assessment, and curriculum development, showing the program's potential to advance faculty DEI-competences. |  |

**References - Appendix**

1. Boor K, Van Der Vleuten C, Teunissen P, Scherpbier A, Scheele F. Development and analysis of D-RECT, an instrument measuring residents’ learning climate. Medical Teacher. 2011;33(10):820-7.

2. Trullàs JC, Blay C, Sarri E, Pujol R. Effectiveness of problem-based learning methodology in undergraduate medical education: a scoping review. BMC medical education. 2022;22(1):104.

3. Karp NC, Hauer KE, Sheu L. Trusted to learn: a qualitative study of clerkship students’ perspectives on trust in the clinical learning environment. Journal of General Internal Medicine. 2019;34:662-8.

4. Cox T. Cultural diversity in organizations: Theory, research and practice: Berrett-Koehler Publishers; 1994.

5. Carter P, Darling-Hammond L. Teaching diverse learners. Handbook of research on teaching. 2016:593-638.

6. Lee KB, Vaishnavi SN, Lau SK, Andriole DA, Jeffe DB. Cultural competency in medical education: demographic differences associated with medical student communication styles and clinical clerkship feedback. Journal of the National Medical Association. 2009;101(2):116-26.

7. Bullock JL, Lockspeiser T, Del Pino-Jones A, Richards R, Teherani A, Hauer KE. They Don't See a Lot of People My Color: A Mixed Methods Study of Racial/Ethnic Stereotype Threat Among Medical Students on Core Clerkships. Acad Med. 2020;95(11S Association of American Medical Colleges Learn Serve Lead: Proceedings of the 59th Annual Research in Medical Education Presentations):S58-s66.

8. Roberts L. Belonging, Respectful Inclusion, and Diversity in Medical Education. Academic medicine : journal of the Association of American Medical Colleges. 2020;95:661-4.

9. Diaz T, Navarro JR, Chen EH. An Institutional Approach to Fostering Inclusion and Addressing Racial Bias: Implications for Diversity in Academic Medicine. Teaching and Learning in Medicine. 2020;32(1):110-6.

10. Wusu MH, Tepperberg S, Weinberg JM, Saper RB. Matching Our Mission: A Strategic Plan to Create a Diverse Family Medicine Residency. Fam Med. 2019;51(1):31-6.

11. Cotton K, Green DON. Leveraging New Media as Social Capital for Diversity Officers: A How-To Guide for Equity, Diversity, and Inclusion Professionals Seeking to Use Social Media to Carve a Niche in the Social Networking Space. In: Benson V, Morgan S, editors. Cutting-Edge Technologies and Social Media Use in Higher Education. Hershey, PA, USA: IGI Global; 2014. p. 294-319.

12. Kolluru S, Wanat MA, Ficzere CH, Akiyode O, Haber SL, Hayatshahi A, et al. Review of Best Practices for Diversity, Equity, and Inclusion Committees Within Colleges of Pharmacy. Am J Pharm Educ. 2023;87(4):ajpe9043.

13. Munn F. Reporting racism on the wards. BMJ. 2017;359:j5178.

14. Regenmortel T. Empowerment als uitdagend kader voor sociale inclusie en moderne zorg. Journal of Social Intervention : Theory and Practice. 2009;18.

15. Rask K, Bailey E. Are Faculty Role Models? Evidence from Major Choice in an Undergraduate Institution. Journal of Economic Education. 2002;33:99-124.

16. Haeger H, Fresquez C. Mentoring for Inclusion: The Impact of Mentoring on Undergraduate Researchers in the Sciences. Cell Biology Education. 2016;15:ar36-ar.

17. Allis L. Just-in-time Learning Best Practices: What it is and isn't. EdMedia + Innovate Learning 2020; Online, The Netherlands: Association for the Advancement of Computing in Education (AACE); 2020. p. 825-8.

18. Lupton KL, O'Sullivan PS. How Medical Educators Can Foster Equity and Inclusion in Their Teaching: A Faculty Development Workshop Series. Acad Med. 2020;95(12S Addressing Harmful Bias and Eliminating Discrimination in Health Professions Learning Environments):S71-s6.
